# Influence of partial k-space filling on the quality of magnetic resonance images*

**DOI:** 10.1590/0100-3984.2015.0028

**Published:** 2016

**Authors:** Tiago da Silva Jornada, Camila Hitomi Murata, Regina Bitelli Medeiros

**Affiliations:** 1 MD, MSc, Physicist, Doctoral Student in Clinical Radiology, Department of Diagnostic Imaging, Escola Paulista de Medicina da Universidade Federal de São Paulo (EPM-Unifesp), São Paulo, SP, Brazil.; 2 Physicist, Hospital São Paulo, São Paulo, SP, Brazil.; 3 Associate Professor Emeritus, Escola Paulista de Medicina da Universidade Federal de São Paulo (EPM-Unifesp), São Paulo, SP, Brazil.

**Keywords:** Magnetic resonance imaging, K-space, Quality control, *In vivo* magnetic resonance imaging

## Abstract

**Objective:**

To study the influence that the scan percentage tool used in partial k-space acquisition has on the quality of images obtained with magnetic resonance imaging equipment.

**Materials and Methods:**

A Philips 1.5 T magnetic resonance imaging scanner was used in order to obtain phantom images for quality control tests and images of the knee of an adult male.

**Results:**

There were no significant variations in the uniformity and signal-to-noise ratios with the phantom images. However, analysis of the high-contrast spatial resolution revealed significant degradation when scan percentages of 70% and 85% were used in the acquisition of T1- and T2-weighted images, respectively. There was significant degradation when a scan percentage of 25% was used in T1- and T2-weighted *in vivo* images (*p* ≤ 0.01 for both).

**Conclusion:**

The use of tools that limit the k-space is not recommended without knowledge of their effect on image quality.

## Abstract (pt)

**Objetivo:**

Estudar a influência da ferramenta scan percentage, usada para a aquisição parcial do espaço K na qualidade de imagens obtidas por ressonância magnética.

**Materiais e Métodos:**

Foi usado um equipamento de ressonância magnética Philips de 1,5 T para obter imagens de um objeto simulador e imagens *in vivo* do joelho de um adulto do sexo masculino.

**Resultados:**

Com o objeto simulador, não ocorreram variações significativas quanto à avaliação da uniformidade e razão sinal-ruído. Entretanto, na análise da resolução espacial de alto contraste, houve significativa degradação nas imagens quando adotada a *scan percentage* a 70% e 85% em T1 e T2, respectivamente. Nas imagens *in vivo* comprovou-se a degradação somente quando adotada a *scan percentage* a 25% (p ≤ 0,01) em T1 e T2.

**Conclusão:**

Não é aconselhável adotar ferramentas que limitem o espaço K sem que haja conhecimento sobre sua influência na qualidade da imagem clinicamente gerada.

## INTRODUCTION

One way of reducing image acquisition time is to reduce the number of lines to be filled in the k-space. However, adopting this measure can have a negative effect on image quality^(1)^.

All of the information used in presenting magnetic resonance imaging (MRI) scans is first acquired in the k-space, which represents the spatial frequency domain^(2,3)^. The k-space is not a physical location in the MRI equipment but an abstract concept that can be understood as a matrix containing a series of data related to the frequencies and phases of the signals collected^(4)^. Whenever an echo-phase encoding plus frequency encoding-is obtained, the information is stored on a line in the k-space. A point in the k-space does not correspond to a point in the image. The peripheral lines contain information on the spatial resolution of the object, whereas the central lines represent the contrast, and the relationship between the k-space and the image can be obtained by the two-dimensional (2D) Fourier transform^(1,2,4)^.

Because k-space filling can be manipulated by the operator, understanding of the concept can redirect the clinical routine^(2,5)^. Different techniques, such as fast spin-echo imaging, parallel image acquisition, keyhole imaging, single-shot imaging, echo-planar imaging, partial echo acquisition, and half-Fourier acquisition^(6)^, organize the collected data in different ways in the k-space^(7)^.

In some models of their MRI equipment, the manufacturer Philips offers a tool called scan percentage (ScP). This resource manipulates k-space filling using a technique similar to the half-Fourier method. What differentiates the ScP tool is that when adopting a protocol with maximum ScP value (100%), all signals are transported and stored in the lines of the k-space without any kind of processing (raw data). However, by changing the ScP value in the acquisition protocol (i.e., adopting a percentage that is lower than the maximum value), some data are not transported, resulting in unfilled lines, specifically the upper and lower peripheral lines. In those regions, the intensity value of each pixel is approximately zero^(8)^. With the half-Fourier method, the individual pixel intensity value is approximately zero only on the lower peripheral lines^(6)^.

Using the ScP tool without prior knowledge of its influence on image quality can compromise the analysis of certain diseases, such as cartilage disorders^(5)^.

The most frequent disease related to hyaline cartilage degeneration is osteoarthritis, for which radiologists have adopted the Kellgren-Lawrence (KL) grading scale used to evaluate the cartilage condition^(9)^. The KL scale uses five grades to indicate the severity of the disease: grade 0 indicates normal cartilage; grade I indicates inconclusive evidence of joint space narrowing; grade II indicates possible cartilage narrowing; grade III indicates visible cartilage narrowing; and grade IV indicates marked cartilage narrowing^(9-11)^.

One of the ways of evaluating MRI is to use reference phantoms^(12)^ and compare technical parameters, although there are not many studies that have associated such images with *in vivo* tests.

It is generally recommended that, for quality control tests on MRI scanners, reference phantoms be used in accordance with guidelines established by international organizations. The specialized literature includes publications from the American Association of Physicists in Medicine^(13)^, the National Electrical Manufacturers Association^(14)^, the American College of Radiology^(15)^, the study of Wood et al.^(16)^, and the Institute of Physics and Engineering in Medicine^(17)^. The American College of Radiology has proposed an MRI Accreditation Program^(15)^. There are no standard practices for quality control tests in Brazil. However, there is a program created by the Brazilian National Accreditation Organization^(18)^ for the evaluation and certification of health services, and there is the Magnetic Resonance Imaging Quality Program created by the Brazilian College of Radiology and Diagnostic Imaging^(19)^. In addition, the study conducted by Mazzola et al.^(20)^ has been adopted as a reference in the field.

The purpose of this study was to evaluate the influence of the ScP tool on image quality, by comparing phantom and in vivo images.

## MATERIALS AND METHODS

### Phantom

The MRI equipment used was the ACS-NT Gyroscan 1.5 T scanner (Philips Medical Systems; Best, the Netherlands) with a 15 mT gradient, and a Magphan^®^ phantom (The Phantom Laboratory, Greenwich, NY, USA) filled with demineralized water, and the signal was captured with a quadrature head coil.

The technical parameters of the protocols adopted in the imaging process with the phantom were as follows: for axial T1-weighted images-field of view (FOV): 230 mm; reduced field of view (RFOV): 100%; repetition time/echo time (TR/ TE): 638/14 ms; flip angle: 90º; number of excitations (NEX): 2; reconstruction matrix: 512 × 512; acquisition matrix: 256 × 256; interslice gap: 1 mm; slice thickness: 4 mm; spacing: 4.4 mm-and for axial T2-weighted images- FOV: 230 mm; RFOV: 100%; TR/TE: 4986/100 ms; flip angle: 90º; NEX: 2; reconstruction matrix: 512 × 512; acquisition matrix: 256 × 256; interslice gap: 1 mm; slice thickness: 4 mm; spacing: 4.4 mm. For each protocol, four ScP variations were studied: 50%, 70%, 85%, and 100%.

The influence that signal intensity variations in the k-space had on image quality was analyzed from phantom images according to three quality criteria-1) uniformity; 2) signal-to-noise ratio (SNR); 3) high-contrast spatial resolution-following the recommendations of the American Association of Physicists in Medicine^(13)^ and the manufacturer's instructions^(21)^.

*1. Uniformity* - Quantifies the performance of the equipment in representing a homogenous region in the image, with a minimum of variation in intensity. Uniformity can be given by the following equation:

$$U=\left[1-\frac{{\bar{S}}_{\max}-{\bar{S}}_{\min}}{{\bar{S}}_{\max}+{\bar{S}}_{\min}}\right].100$$

where *S_max_* is the pixel intensity value with the strongest signal and *S_min_* is the pixel intensity value with the lowest signal.

*2. SNR* - Quantifies the signal fluctuation at a given region of interest. The SNR can be obtained by the following equation:

$$\mathrm{RSR}=\frac{\sqrt{2}\bar{S}}{\sigma }$$

where *S_max_* is the mean signal in a given region of interest and s is the standard deviation resulting from the subtraction of two images.

**Figure 1:**
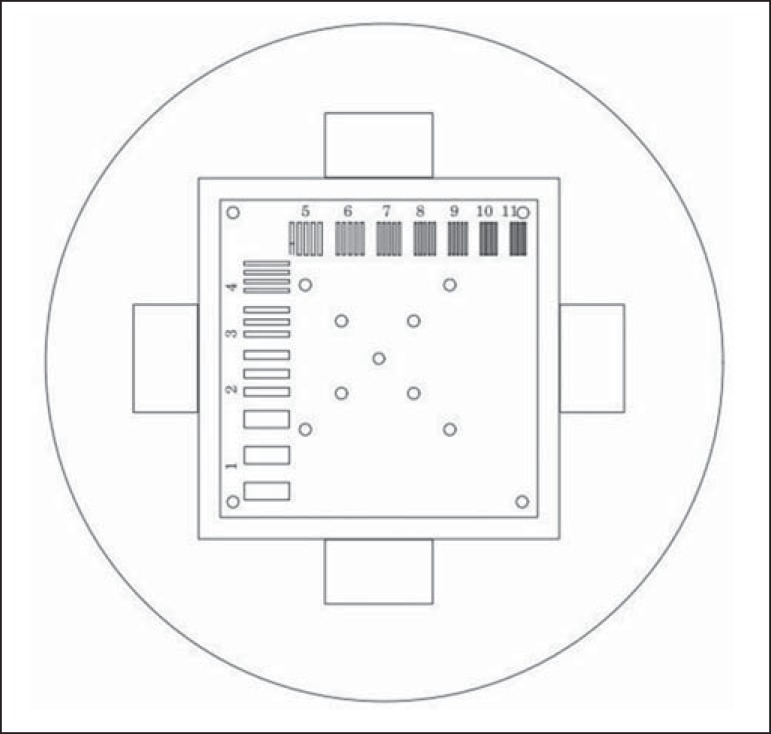
Region of the phantom where the high-contrast spatial resolution analysis is made.

*3. High-contrast spatial resolution* - Shows the capacity of the equipment to distinguish the spacing between objects, without superimposing the structures. It is obtained by a qualitative analysis of the phantom's internal structures on high-resolution images. Internally, the objects represent 1, 2, 3, 4, 5, 6, 7, 8, 9, 10, and 11 pairs of lines/cm (Figure 1).

### In vivo

**Figure 2:**
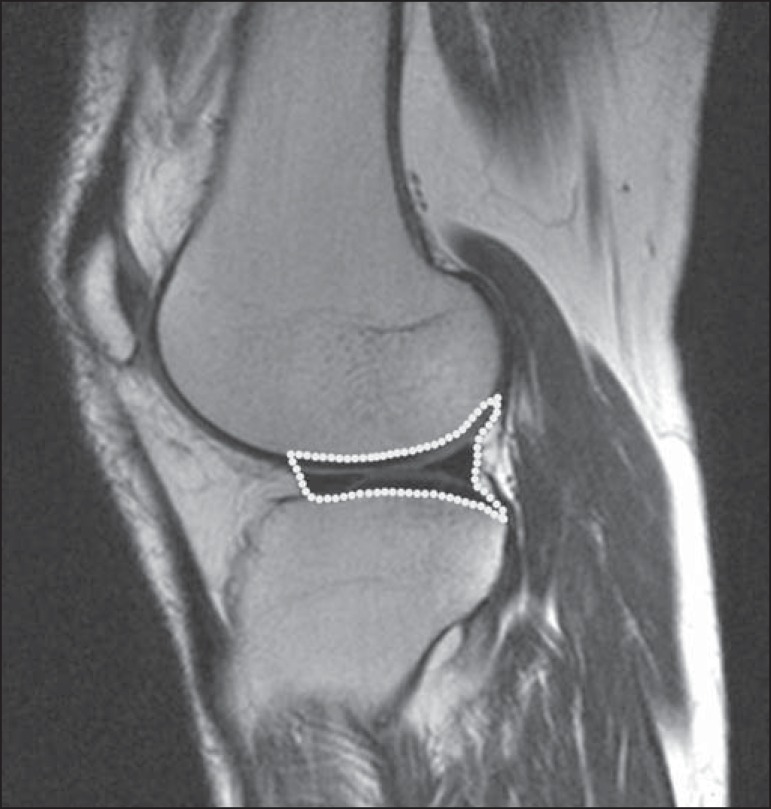
Magnetic resonance imaging scans of the *in vivo* object of study. The white outline indicates the region of analysis.

For the *in vivo* tests, which were approved by the Research Ethics Committee of the Escola Paulista de Medicina - Universidade Federal de São Paulo, the same MRI equipment was used with an appropriate quadrature knee coil, with the following acquisition parameters: for sagittal T1-weighted images-FOV: 230 mm; RFOV: 100%; TR/TE: 535/12 ms; flip angle: 90º; NEX: 2; reconstruction matrix: 512 × 512; acquisition matrix: 256 × 256; interslice gap: 1 mm; slice thickness: 4 mm; spacing: 4.4 mm-and for sagittal T2-weighted images-FOV: 230 mm; RFOV: 100%; TR/TE: 2440/60 ms; flip angle: 90º; NEX: 2; reconstruction matrix: 512 × 512; acquisition matrix: 256 × 256; interslice gap: 1 mm; slice thickness: 4 mm; spacing: 4.4 mm. For each protocol, we studied seven ScP variations (25%, 40%, 50%, 60%, 70%, 85%, and 100%), and we used the RadiAnt DICOM Viewer software (Medixant, Poznan, Poland) to analyze the images^(22)^, as shown in Figure 2.

### Statistical analysis

Initially, a normal distribution of the pixel intensity values in the region of the hyaline cartilage was observed (Figure 2) with the Kolmogorov-Smirnov Z test. After having verified the normality of the data, we performed analysis of variance with the Student-Newman-Keuls post hoc test. For non-normal data, we opted to use the Kruskal-Wallis test with the Müller-Dunn post hoc test. The statistical analysis software used were the SPSS Statistics software package (IBM Corp., Armonk, NY, USA) and BioEstat, version 5.3 (Instituto Mamirauá, Tefé, Brazil).

## RESULTS

**Figure 3:**
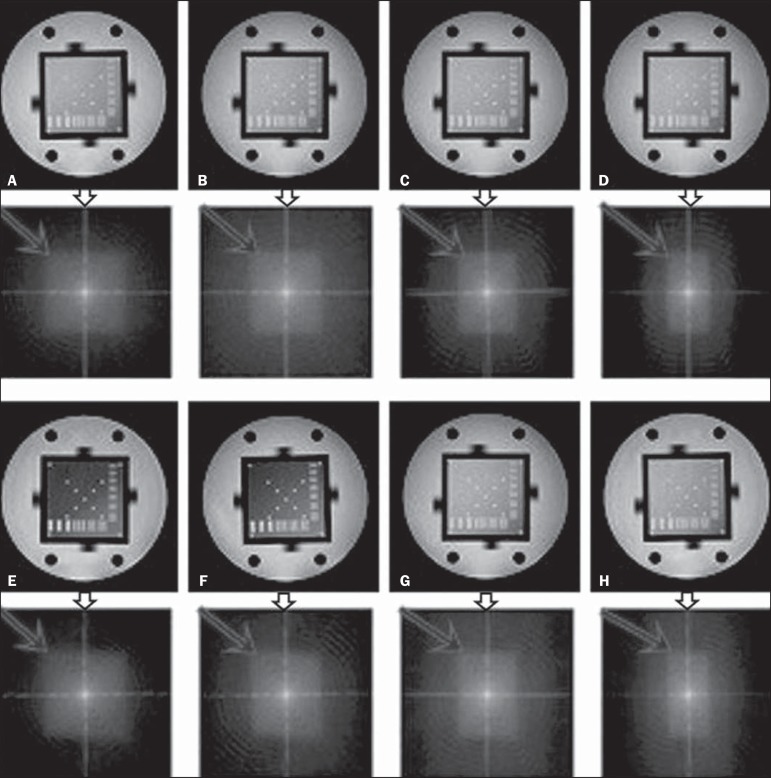
Phantom images with the following ScP variations: 100% (**A**), 85% (**B**), 70% (**C**) and 50% (**D**), T1-weighted; 100% (**E**), 85% (**F**), 70% (**G**), and 50% (**H**), T2-weighted. The white arrows indicate the k-space of the respective images.

When analyzing the phantom images, with help of software MatLab^®^ (MathWorks; Natick, Mass., USA), we applied the inverse 2D Fourier transform in the images obtained with each ScP variation, resulting in the k-space of the respective image, as shown in Figure 3.

The influence of signal intensity variation in the k-space, in relation to uniformity, SNR, and high-contrast spatial resolution analysis, is shown in Table 1.

**Table 1:** Values for analyzing the quality parameters.

	T1-weighted					T2-weighted			
	Scan percentage					Scan percentage			
Parameters	50%	70%	85%	100%		50%	70%	85%	100%
Uniformity (%)	92	94	90	94		94	95	94	96
Signal-to-noise ratio	64	65	66	66		153	169	167	168
High-contrast spatial	2	4	5	5		2	4	4	5
resolution (pl/mm)									

**Figure 4:**
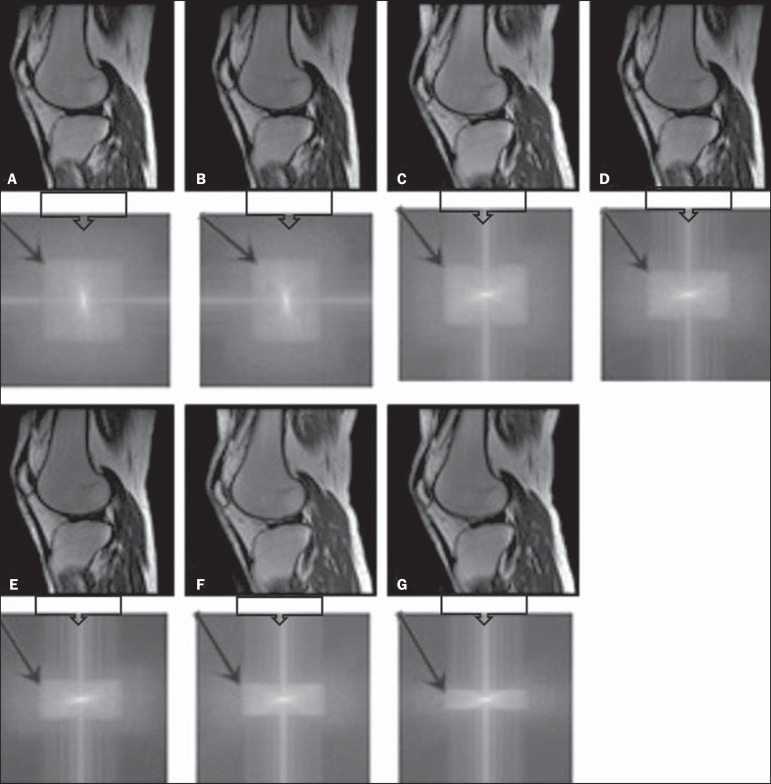
T1-weighted magnetic resonance imaging scans with the following ScP variations: 100% (**A**), 80% (**B**), 70% (**C**), 60% (**D**), 50% (**E**), 40% (**F**), and 25% (**G**). The black arrows indicate the k-space of the respective images.

**Figure 5:**
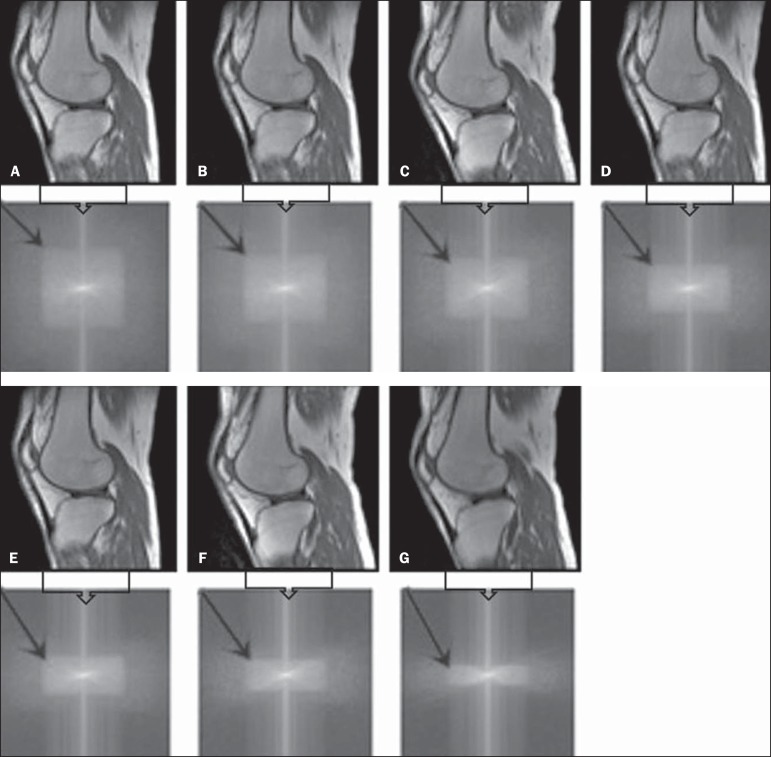
T2-weighted magnetic resonance images with the following ScP variations: 100% (**A**), 80% (**B**), 70% (**C**), 60% (**D**), 50% (**E**), 40% (**F**), and 25% (**G**). The black arrows indicate the k-space of the respective images.

Adopting seven percentages of k-space filling (25%, 40%, 50%, 60%, 70%, 85%, and 100%), we obtained *in vivo* anatomical images (Figures 4 and 5).

The Kolmogorov-Smirnov Z test of normality showed *p* ≤ 0.0001 in all regions studied, regardless of the ScP variation chosen, proving that the pixel intensity values represents a parametric (normal) distribution. Therefore, the difference in pixel value intensity between the different ScP variations was determined with analysis of variance and the Student-Newman-Keuls post hoc test, both at a 5% level of significance.

Table 2 shows the results of the statistical analysis of T1-weighted images, and Table 3 shows the results of the statistical analysis of T2-weighted images.

**Table 2:** Statistical analysis results of the T1-weighted images.

	85%	75%	60%	50%	40%	25%
Minimum pixel intensity	3	44	40	74	74	54
Maximum pixel intensity	63	4	6	5	3	4
Difference in variance in relation to the image at an ScP of 100%	1.550	1.500	8.700	11.5026	11.250	22.700
*P*-value	Not statistically perceptible	Not statistically perceptible	Not statistically perceptible	Not statistically perceptible	Not statistically perceptible	*p* ≤ 0.01

**Table 3:** Statistical analysis results of the T2-weighted images.

	85%	75%	60%	50%	40%	25%
Minimum pixel intensity	0	0	0	0	0	0
Maximum pixel intensity	169	74	47	83	97	62
Difference in variance in relation to the	2.9615	5.2692	6.0769	6.6538	7.6154	14.0769
image at an ScP of 100%						
*P-value*	Not statistically perceptible	Not statistically perceptible	Not statistically perceptible	Not statistically perceptible	Not statistically perceptible	*p* ≤ 0.01

## DISCUSSION

Through visual analysis of each k-space image, it is possible to identify a central area with greater signal intensity. As the k-space filling percentage is reduced, a gradual loss in intensity is observed, regardless of the weighting adopted. With an ScP variation of 50%, the signal loss is more pronounced, because half of the k-space is omitted.

The values associated with uniformity in the image do not show significant variation with the ScP values adopted, remaining within the > 90% acceptance limit^(15,23)^. For the evaluation of SNR, a comparison with the reference values provided by the manufacturer, using serial measurements, is recommended. Because those data are unavailable, we stipulated an acceptance margin of ± 10% in SNR variation in relation to the acquired value with 100% ScP . For T1weighted images, the SNR varied by approximately 1%. For T2-weighted images, the greatest SNR variation was 9.8% (when an ScP variation of 50% was adopted). Therefore, the SNR is in conformity with the variation adopted. Because this filling method leaves the central k-space lines unaltered, it was possible to the keep the uniformity and SNR values in conformity with the criteria adopted.

In the spatial resolution analysis, there was a loss in image quality when ScP variations of 70% and 85% in T1- and T2-weighted images, respectively. The recommended spatial resolution is > 1 mm (5 pl/mm) with well-defined borders between the phantom test structures^(15,23)^, a criterion that was not met with ScP variations of 70% and 85% in T1- and T2weighted images, respectively. An omission of 25% of the peripheral lines in the k-space proved to be sufficient to cause significant degradation of the high-contrast spatial resolution.

The cartilage studied was classified as grade 0 at all ScP variations. There were no changes in the KL scale cartilage grade in T1- or T2-weighted images. However, the visual analysis of all k-spaces (Figures 4 and 5) showed a reduction in the signal intensity, resulting in a loss of contrast and structure resolution.

According to Albuquerque et al.^(11)^, the analysis of cartilage degradation and its KL scale grading are subjective and can be influenced by the level of experience of the radiologist. The loss of contrast and spatial resolution detected in the images can lead to misdiagnoses by inexperienced professionals.

On the T1- and T2-weighted images, the results were p ≤ 0.01, with evidence to reject the null hypothesis. However, there was at least one group with nonhomogeneous population variations. Therefore, partial k-space filling showed a statistically significant variation in the pixel intensity values in at least one of the adopted situations in relation to an ScP value of 100%.

With the Student Newman-Keuls post hoc test, it was proven that the variation in k-space filling at an ScP variation of 25% showed a statistically significant difference in relation to that observed at an ScP variation of 100% in the T1- and T2-weighted images.

## CONCLUSION

The phantom images showed variations in high-contrast spatial resolution that were not perceptible in the *in vivo* images, because the phantom contains known standards that allow a more careful analysis.

The analysis involving the phantom showed that it is possible to use ScP variations of 70% and 85% in the acquisition of T1- and T2-weighted clinical images, respectively, without significant quality loss. Adopting values below those acquisition levels would require analysis by a multidisciplinary team and involving a significant sample of patients.

The use of tools that limit the k-space is not recommended without knowledge of their effect on image quality.
